# Replication Fork Remodeling and Therapy Escape in DNA Damage Response-Deficient Cancers

**DOI:** 10.3389/fonc.2020.00670

**Published:** 2020-05-05

**Authors:** Martin Liptay, Joana S. Barbosa, Sven Rottenberg

**Affiliations:** ^1^Institute of Animal Pathology, Vetsuisse Faculty, University of Bern, Bern, Switzerland; ^2^Bern Center for Precision Medicine, University of Bern, Bern, Switzerland

**Keywords:** DNA replication, replication fork, chemotherapy, drug resistance, DNA damage response, DNA damage tolerance, PARP inhibitors, BRCA1/2

## Abstract

Most cancers have lost a critical DNA damage response (DDR) pathway during tumor evolution. These alterations provide a useful explanation for the initial sensitivity of tumors to DNA-targeting chemotherapy. A striking example is dysfunctional homology-directed repair (HDR), e.g., due to inactivating mutations in *BRCA1* and *BRCA2* genes. Extensive efforts are being made to develop novel targeted therapies exploiting such an HDR defect. Inhibitors of poly(ADP-ribose) polymerase (PARP) are an instructive example of this approach. Despite the success of PARP inhibitors, the presence of primary or acquired therapy resistance remains a major challenge in clinical oncology. To move the field of precision medicine forward, we need to understand the precise mechanisms causing therapy resistance. Using preclinical models, various mechanisms underlying chemotherapy resistance have been identified. Restoration of HDR seems to be a prevalent mechanism but this does not explain resistance in all cases. Interestingly, some factors involved in DNA damage response (DDR) have independent functions in replication fork (RF) biology and their loss causes RF instability and therapy sensitivity. However, in BRCA-deficient tumors, loss of these factors leads to restored stability of RFs and acquired drug resistance. In this review we discuss the recent advances in the field of RF biology and its potential implications for chemotherapy response in DDR-defective cancers. Additionally, we review the role of DNA damage tolerance (DDT) pathways in maintenance of genome integrity and their alterations in cancer. Furthermore, we refer to novel tools that, combined with a better understanding of drug resistance mechanisms, may constitute a great advance in personalized diagnosis and therapeutic strategies for patients with HDR-deficient tumors.

## DNA Damage Response-Targeted Cancer Therapy and Resistance

Damage to DNA occurs naturally in cells during cellular metabolism, or after exposure to external agents such as ultraviolet light, ionizing irradiation (IR), or genotoxic chemicals (1). While healthy cells are able to repair the DNA lesions, cells that have defects in the DNA damage response (DDR) pathway do not repair the lesions as efficiently, resulting in genome instability and potentially the development of cancer (2). Instructive examples of malignancies with defects in the DDR are ovarian and breast cancers with mutations in genes of the homologous recombination (HR) pathway, such as *BRCA1* and *BRCA2* (3–7). The HR pathway is one of the three major cellular pathways that repair DNA double strand breaks (DSBs) (8–10). Whereas, the other pathways, classical non-homologous end-joining (NHEJ) and theta-mediated end joining (TMEJ) do not require a template for repair and tend to be error-prone, HR occurs after DNA replication and uses the undamaged sister chromatid as a template for error-free repair of DSBs [reviewed in (9, 11)].

Although DDR alterations cause mutagenesis and malignant transformation, they also provide a therapeutic opportunity that can be explored by DNA damage-inducing therapies (12, 13). In fact, alterations in the DDR even provide a useful explanation for the initial drug sensitivity. Most cancers have lost a critical DDR pathway during cancer evolution (14, 15). Patients therefore respond to clinical interventions that cause DNA damage, e.g., chemotherapy using DNA crosslinkers and radiotherapy. Whereas, the normal cells of the body can still cope with the damage, the tumor cells that lack proper DNA repair cannot and die. Accordingly, HR-deficient cancers (e.g., due to *BRCA1/2* mutations) are often sensitive to classical DNA-crosslinking agents such as platinum-based drugs (13, 16). However, these agents are associated with significant side effects due to the damage of normal tissues (17).

An alternative to this conventional therapy is a more targeted type of treatment that is based on the synthetic lethality concept: the mutation in one of two genes is harmless for the cells but the simultaneous inactivation of those two genes is lethal (18, 19). Because tumors that have lost a certain DDR pathway rely more on other DNA repair mechanisms, selectively inhibiting these alternative pathways gives an opportunity to induce synthetic lethality in these tumor cells. In contrast, the normal cells still have all DDR pathways available and can cope with the damage induced by the treatment.

A successful example of this concept is the approval of poly(ADP)ribose polymerase (PARP) inhibitors (PARPi) to target BRCA1/2-deficient ovarian and breast cancers (20, 21), with relatively moderate side effects [reviewed in (22, 23)]. Several PARP enzymes, and in particular its founding member PARP1, are important in coordinating responses to DNA damage (24, 25). PARP1 is quickly recruited to single-stranded DNA (ssDNA) sites upon damage and catabolizes the formation of branched PAR polymers, which then serve as a scaffold for the recruitment of downstream repair factors (26). When the lesion is removed, poly(ADP-ribose) glycohydrolase (PARG) removes the PAR chains and PARP1 is released from DNA, together with the other involved proteins. PARPi inhibit the PARylation reaction and trap PARP to DNA, delaying the repair of the damage. It is thought that accumulation of SSBs in the absence of PAR synthesis and physical trapping of PARP1 on DNA eventually lead to RF collapse and DSBs (8, 27, 28). Since PARP1 also senses unligated Okazaki fragments during DNA replication and facilitates their repair, the synthetic lethality may also origin from replication-associated single-stranded DNA gaps (29). Recently, another model for PARPi-induced genotoxicity was presented, where PARPi deregulates restart of transiently stalled forks (see “Replication fork reversal and its players” below), elevating the fork progression rate above a tolerable threshold in the presence of DNA damage (30–32). However, the relevance of the mechanisms mentioned above in different model systems and different therapy contexts remains to be better understood. Importantly, since HR is required for error-free DSB repair following replication, BRCA1/2-deficient tumor cells lacking HR activity are not able to tolerate the damage induced by PARPi and they eventually die, whereas normal cells can cope with PARPi treatment (27).

Despite the clinical benefits of PARPi, most patients with disseminated BRCA1/2-mutated cancer still die because their tumors either show upfront resistance or develop secondary resistance (33). Thus, drug resistance remains a major challenge in targeting DDR pathways.

Mechanisms of resistance to PARPi in HR-deficient tumors have been studied extensively in preclinical models [reviewed in (34)]. Residual hypomorphic activity or reactivation of BRCA1/2 function by secondary mutations, is one of the major mechanisms found in patients (5, 35–39). Moreover, the restoration of HR independently of BRCA1 function (via the downregulation of factors involved in blocking DNA end resection and promoting NHEJ) is also prominent in animal models (40–54) and we expect that this also occurs in humans. Additional mechanisms discovered are related to the upregulation of the drug efflux transporter ABCB1/P-gp (55, 56), the loss of the drug target via downregulation of PARP1 in BRCA1/2-proficient cells (57), PARP1 point mutations that abrogate PARPi-induced trapping (58), or the partial restoration of PARylation activity via the loss of PARG, the functional antagonizer of PARP1 (59).

More recently, attention has been brought to the contribution of replication fork (RF) integrity to genome stability and drug response (60, 61). Interestingly, besides their role in DNA repair, BRCA1/2 are also important to protect stalled RFs, allowing the resolution of replication intermediates while preventing excessive nucleolytic degradation (62–64). This dual role of BRCA1/2 in DNA repair and RF protection makes BRCA1/2-deficient cells highly sensitive to DNA damaging agents and drugs affecting replication (see more details in the section “Fork stability as a resistance mechanism in BRCA-deficient tumors”). Besides BRCA1/2, other DNA repair factors such as RNF8, RNF168, 53BP1, and RAD51 are present at RFs and play a role in their dynamics (65–70). In agreement with this, several studies have demonstrated that restored stability of RF in BRCA1/2-deficient cells achieved via re-activation of BRCA1/2 or additional loss of other factors regulating RF processing, confers resistance to PARPi and platinum drugs (62, 63, 71–73) [reviewed in (60, 61)].

Hence, various well-known mediators of DSB repair have independent functions in RF biology. Since their defect is linked to increased anti-cancer therapy sensitivity, it raises the question whether the defective RF metabolism is the main determinant of anti-cancer therapy response or, at least, a major contributor.

Given the increasing implications of RF homeostasis for cancer therapy, we focus our attention in this review to RF remodeling and the different methods currently used to study RF constitution and dynamics. Next, we discuss crucial molecular players of these processes and the relation of PARP and PARPi with the RF remodeling “metabolism.” In addition, we discuss the role of fork stability and restart in cancer drug resistance and the biological role of DDT pathways in the maintenance of genome integrity and cancer. Moreover, we will suggest some practical applications of this knowledge in the clinic, in terms of diagnosis and prognosis, predicting personalized treatment responses, and for the development of new therapeutic strategies.

## The Tool-Box to Study RF Structure, Composition and Dynamics

To investigate RF biology, high-resolution, quantitative molecular tools are necessary, in particular for the study of protein interactions at RFs during unperturbed S-phase or replication stress. Because each method has it strengths and pitfalls, a combination of several methods is useful to obtain a complete picture of the hypothesis to be tested. Before focusing on the mechanisms of RF biology in the context of cancer therapy, we provide a brief outline of the most commonly used techniques.

### Electron Microscopy (EM)

Electron microscopes use a beam of accelerated electrons as a source of illumination. Since the wavelength of electrons can be up to 100,000 times shorter than that of visible light photons, electron microscopes have a much higher resolution than light microscopes and are ideal to visualize small structures. Actually, EM is the only method that allows direct observation and quantification of DNA replication intermediates. Several structures, such as reversed forks, Holliday junctions and even the distinction between single-stranded DNA (ssDNA) and double-stranded DNA (dsDNA) have been observed using this method (74).

**Figure 1:**
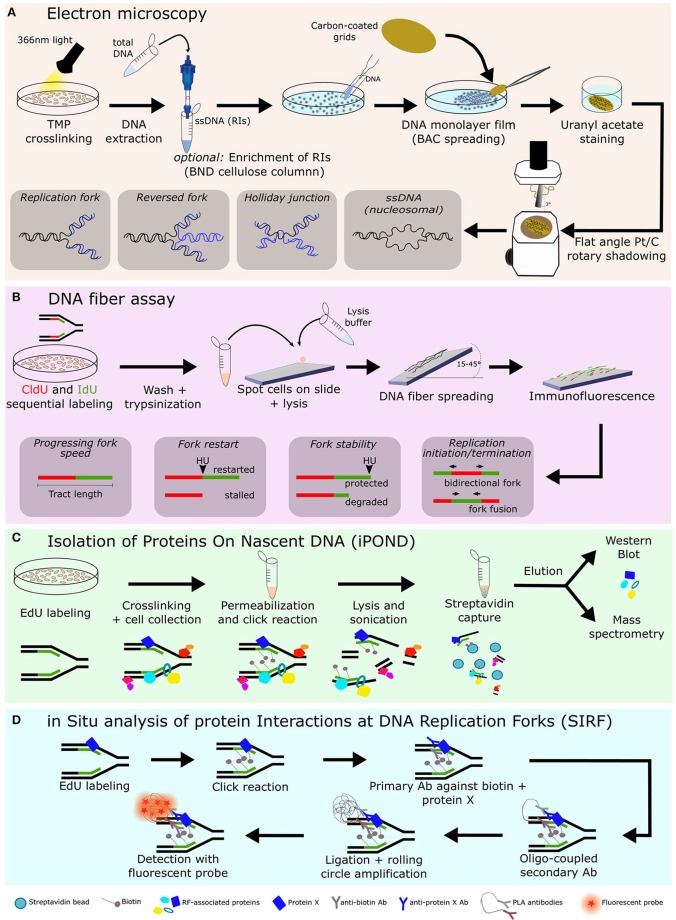
Overview of techniques frequently used to study replication fork biology. Various methodologies, including electron microscopy **(A)**, single molecule DNA fiber assay, using the spreading technique **(B)**, iPOND **(C)**, and SIRF **(D)**, are being used to study replication fork-associated processes. Combining these techniques allowed many research groups to identify novel factors associated with replication forks and their role in replication fork dynamics and replication stress responses. Ab, antibody; BAC, benzyl-dimethyl- alkylammonium chloride; BND, benzoylated-naphthoylated DEAE; CldU, chlorodeoxyuridine; EdU, 5-Ethynyl-2′-deoxyuridine; IdU, iododeoxyuridine; iPOND, isolation of proteins on nascent DNA; PLA, proximity ligation assay; Pt/C, Platinum/carbon; RF, Replication fork; RIs, replication intermediates; SIRF, *in situ* analysis of protein interactions at DNA replication forks; TMP, tri-methyl-psoralen.

Briefly, living cells are exposed to tri-methyl-psoralen (TMP) and irradiated with 365–366 nm monochromatic light to cross-link DNA. This crosslinking step preserves DNA replication intermediate (RI) structures during the subsequent extraction and enrichment procedures. Genomic DNA is then extracted and, in an optional step, RI are enriched by binding, washing and elution in a benzoylated-naphthoylated DEAE (BND) cellulose column, since this resin has high affinity to ssDNA (which is always present at RFs). Afterwards, the DNA sample is concentrated in size-exclusion columns and spread in the presence of the cationic detergent benzyl-dimethyl- alkylammonium chloride (BAC). This monolayer of DNA is absorbed to carbon-coated grids and stained with uranyl acetate. Finally, the individual DNA molecules can be visualized after the grids undergo flat angle rotary shadowing with platinum (74) (Figure 1A).

The high resolving power of EM (in the range of 30–50 base-pairs) allows the visualization of the fine architecture of DNA structures, such as reversed forks, and, combined with drug treatment or genetic manipulations, can reveal any kind of DNA alterations caused by these perturbations. Moreover, because nucleosomal DNA is not accessible to the crosslinking reagent psoralen, the final, deproteinized DNA will appear as ssDNA bubbles that represent the nucleosome position *in vivo*, providing valuable information on the chromatin organization on replicating DNA (74) (Figure 1A). Despite the enormous benefits of EM, it is a relatively laborious technique, it requires specialized, expensive equipment and it is a static method that only provides a snapshot of the RIs at a given time-point (Table 1).

**Table 1:** Summary of the advantages and disadvantages of the different techniques used to study replication fork biology.

**Technique**	**Advantages**	**Disadvantages**
Electron microscopy	○ Direct visualization and quantification of fork structures ○ High resolution: 30–50 base pairs	○ Static method ○ Laborious and requires specialized and expensive technique and equipment
DNA fiber assay	○ Single molecule resolution ○ Can measure several parameters: rate of fork elongation, inter-origin distances, frequency of origin firing, and frequency of fork collapse ○ Allows monitoring the dynamics of replication perturbation for a prolonged period of time	○ Relatively low resolution (only length differences corresponding to at least 2–4 Kb of DNA can be observed) ○ Inter-observer variability of the image analysis
iPOND	○ Improved sensitivity (compared to IF) ○ Combined with pulse-chase methods provides high spatial and temporal resolution of protein dynamics. ○ Allows analysis of posttranslational modifications ○ Compatible with unbiased screening approaches. ○ Coupling with SILAC/mass spectrometry: highly quantitative and unbiased	○ Laborious ○ Large amount of starting material required ○ Limited quantification potential ○ Does not consider heterogeneity of cell populations ○ SILAC/mass spectrometry: requires high-cost specialized equipment with limited access
SIRF	○ Single cell resolution ○ Allows analysis of heterogeneous cell populations (location and type) ○ Readily quantifiable ○ Sensitive (very little starting cell material) ○ Does not require special equipment	○ Not all epitopes at the forks may be accessible to antibodies ○ Limited to distances no >~40 nm

*IF, immunofluorescence; iPOND, Isolation of proteins on nascent DNA; SILAC, stable isotope labeling with amino acids in cell culture; SIRF, in situ analysis of protein interactions at DNA replication forks*.

### DNA Fiber Assay

In this procedure, ongoing replication events are sequentially labeled with two thymidine analogs [commonly iododeoxyuridine (IdU) and chlorodeoxyuridine (CldU)] and, after cell lysis, individual DNA molecules are stretched into fibers using the combing (75, 76) or the spreading technique (represented in Figure 1B) (77). The two modified nucleotides are then detected by two-color immunofluorescence and visualized in a fluorescence microscope (Figure 1B).

Unlike EM, the visualization of individual RFs using the DNA fiber assay provides a better understanding of the dynamic behavior of RFs, based on several parameters, such as: the speed of ongoing RFs, the number of newly initiated forks, the distance between replication origins, the frequency of fork stalling/collapse, for instance, upon induction of replication stress (78, 79). Therefore, the combination of different experimental variables, such as the duration of labeling with thymidine analogs, the existence (or not) and extent of chase after labeling, as well the exposure to different genotoxic agents, gives a global picture of the fluctuating alterations in RFs. Combinations of EM and DNA fiber methods offer optimized conditions to elucidate mechanistic aspects of the cellular responses to specific types of replication stress (80).

The scale of the detected DNA fibers is 1 μm = 2–4 Kb, which means that only RF degradation of at least 2 kb can be directly observed, whereas smaller losses are undetected, making this a technique relatively low in resolution, when compared to others (61) (Table 1). Even though the “simple” DNA fiber assay does not provide information on the location of the RFs in the genome, it can be combined with a DNA probe (Fluorescence *in situ* Hybridization-FISH) specific for a certain genomic region (81). Due to the limited sensitivity of immunofluorescence, the detection of proteins at RFs is not feasible with this method (Table 1).

### Isolation of Proteins on Nascent DNA (iPOND)

As its name indicates, iPOND is an approach focused on the detection of proteins associated with nascent DNA. In this method, cells are incubated with the thymidine analog 5-Ethynyl-2′-deoxyuridine (EdU) to label newly replicated DNA. After cross-linking of proteins and DNA with formaldehyde, the click reaction in performed to link biotin to EdU (82). After cell lysis and sonication to shear chromatin, proteins in close proximity to biotin and EdU-labeled DNA are purified with streptavidin-coated agarose beads. These isolated proteins are then resolved by Western blotting or mass spectrometry (69) (Figure 1C). Besides allowing the identification of proteins at active RFs, this technique also enables the investigation of proteins recruited to stalled and collapsed forks, depending on the addition of different replication stress-inducing agents to the cells (69).

Compared to immunofluorescence, iPOND is a more sensitive technique and also enables the analysis of posttranslational modifications. Additionally, combined with pulse-chase experiments, it offers a high spatial and temporal resolution of protein dynamics at replicating DNA. Another advantage of iPOND is the possibility to combine it with unbiased screening approaches by coupling iPOND to mass spectrometry (Table 1). Hence, this methodology is very useful to identify new proteins present at active and perturbed RFs (69).

Despite its relative high sensitivity, iPOND lacks an amplification step, which means that large amounts of starting material are needed to achieve sufficient protein for detection (82). It is also a laborious and not very trivial technique, requiring specialized technical skills. Other drawbacks of this tool are its limited quantitative potential and the fact that it analyses cells as a whole population, not considering individual cell heterogeneity (Table 1).

One extension of mass spectrometry-coupled iPOND is the combination with stable isotope labeling with amino acids in cell culture (SILAC). For this purpose, two different cell populations are grown in a medium containing either normal amino acids or amino acids labeled with stable non-radioactive heavy isotopes. This way, the abundance of specific proteins can be directly compared and quantified between the two samples (69).

An alternative protocol for iPOND, named aniPOND (accelerated native iPOND) has also been developed. The major advantages of aniPOND compared to the earlier described iPOND are the milder lysis conditions that preserve better the DNA-protein complexes, the absence of the formaldehyde crosslinking step that may interfere with downstream analysis, and an improved protein yield (83).

### *In situ* Analysis of Protein Interactions at DNA Replication Forks (SIRF)

SIRF is a technology that fuses iPOND and a modified version of the proximity ligation assay (PLA), used to detect proteins in close proximity to others (84). In this method, like in iPOND, EdU is incorporated into replicating DNA and then biotinylated using the click chemistry (85). Afterwards cells are incubated with primary antibodies against biotin and the protein of interest and detection follows the principles of PLA: two secondary antibodies conjugated with oligonucleotides are added to the cells and bind to the primary antibodies. When the secondary antibodies (and consequently EdU-labeled DNA and the protein of interest) are in close proximity (<40 nm), the two oligonucleotides can anneal to each other and form a circular DNA structure that serves as a template for a PCR-based amplification reaction (rolling circle amplification). These amplified DNA circles are then detected by sequence-specific DNA fluorescent probes, allowing the visualization and quantification of signal that corresponds to the sites of interaction between active RFs and the protein of interest (85) (Figure 1D). Besides SIRF using EdU to label nascent DNA, mapping proteins at forks can also be assessed by the standard PLA method between any given protein and PCNA (or other fork components).

The combination of this efficient and sensitive tool with other immunofluorescence parameters, such as cell cycle or cell identity markers, enables the analysis of heterogeneous cell populations with a single cell resolution. Additionally, it can be performed in any standard molecular biology laboratory, as it does not require special equipment (85). Pitfalls of SIRF are the fact that only interactions closer than 40 nm can be visualized and that some epitopes at RFs may not be accessible to antibodies (85) (Table 1).

## Replication Fork Reversal and its Players

**Figure 2:**
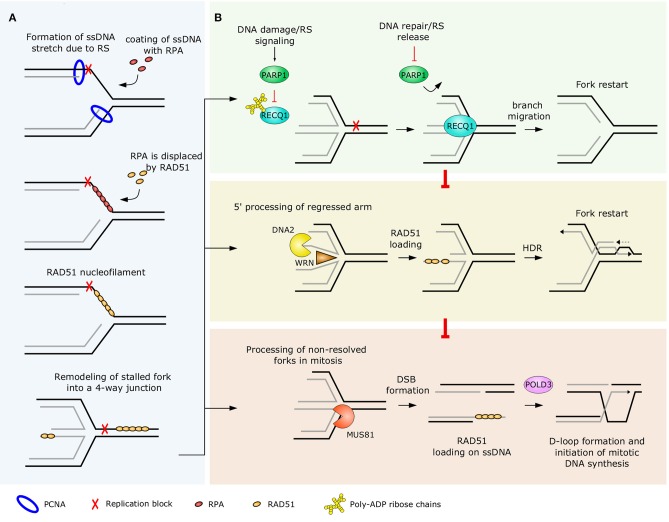
RAD51-mediated RF reversal **(A)** and an overview of replication fork restart mechanisms **(B)**. **(A)** At stalled replication forks, ssDNA tracks are protected and coated with RPA. The DNA recombinase RAD51 replaces RPA and binds to DNA, contributing to the remodeling of the stalled fork into a reversed fork (4-way) structure. Besides RAD51, there are other replication fork remodelers, mentioned in the main text, but for simplicity only RAD51 is represented in this figure. **(B)** PARP1-mediated suppression of RECQ1 helicase is an important regulator of a premature restart of reversed forks (upper panel). Because of the least amount of processing involved, RECQ1-mediated pathway represents the first-choice restart mechanism of reversed forks. DNA2/WRN-driven restart involves regulated processing of the regressed arms and uses HDR to resolve the replication intermediate (middle panel). Reversed forks that could not be restarted in S phase are processed by MUS81 endonuclease later in mitosis and DSB break is formed in the process. The collapsed fork is then rescued by POLD3-driven D-loop formation and synthesis re-initiation (lower panel). PCNA, proliferating cell nuclear antigen; RPA, replication protein A.

Remodeling of RFs involves unwinding of newly synthesized strands and annealing of nascent and parental strands. In this process, the standard three-way junction forks are converted into four-way junction structures. Since annealing of nascent DNA strands form regressed arms at the fork, this remodeling event is called RF reversal (Figure 2A). This was shown to be an effective mechanism allowing cells to cope with replication stress and to maintain genome integrity (70). Interestingly, recent work of Mutreja et al. (86) has demonstrated that replication fork reversal can be regulated globally and may represent a “safety brake” to prevent potential collisions of ongoing unaffected forks with DNA lesions ahead of them. The authors also demonstrated that this global fork slowing and reversal requires ATR-dependent signaling (86).

The initial step of fork reversal is associated with the accumulation of ssDNA at challenged RFs. This can occur either by physical uncoupling of the polymerase and replicative helicase or by controlled nucleolytic digestion of nascent DNA in certain contexts, such as in response to inter-strand crosslinks (ICLs) or increased torsional stress (70, 87). Uncovered ssDNA at the affected fork is promptly recognized by a highly abundant Replication protein A (RPA). The high affinity of RPA toward ssDNA allows a dynamic cellular response to a variety of replication stress-inducing agents of both endogenous and exogenous origin (88–90).

The interaction of RPA with ssDNA is highly dynamic and involves repeated dissociation and re-association of RPA subunits due to conformational changes. Dynamic interaction of RPA with both DNA and other proteins allows RPA to carry out various functions and is crucial for maintaining the stability of the fork affected by replication stress (Figure 2A). First, coating of ssDNA with RPA removes secondary structures (91, 92). RPA nucleofilaments then attract checkpoint signalization proteins such as ATR and its interactor ATRIP (ATR-interacting protein) to initiate a global cellular response to replication stress (89, 93). Furthermore, RPA nucleofilaments help recruit and regulate the activity of various DNA repair proteins required for stabilization and recovery of the challenged fork (94, 95). All these functions are essential for preventing RF collapse and maintenance of chromosomal integrity (91).

RAD51 recombinase is well-known for catalyzing strand-invasion in HR repair of DNA double-strand breaks. Loading to ssDNA at double-strand breaks is highly dependent on its interaction with BRCA2. However, RAD51 also plays an important role in regulating RF reversal (Figure 2A) (70). Interestingly, these two functions are genetically separated, since its recruitment to stalled forks and its enzymatic activity promoting fork reversal are BRCA2-independent (96, 97). Dungrawala et al. (98) identified a ssDNA-binding protein, RADX, to be enriched at RFs and to antagonize the accumulation of RAD51 and RF reversal. Nevertheless, how the recruitment of RAD51 to stalled forks is regulated remains largely elusive. Due to impaired fork reversal, cells depleted of RAD51 do not show reduced RF progression following genotoxic treatments, leading to hypersensitivity to a wide-range of genotoxic agents and increased frequency of chromosome breakage (70).

Several remodelers have been shown to associate with stalled RFs and drive their reversal, such as SMARCAL1, ZRANB3, and HLTF (94, 99, 100). Interestingly, a common feature of all three is the lack of a 3′-ssDNA unwinding activity typical for helicases. Instead, upon recruitment to stalled forks, their critical role in remodeling of challenged RFs is facilitated by their ATP-dependent dsDNA translocase activity, allowing the formation of regressed arms by unwinding of newly synthesized strands and annealing of nascent and parental strands (99, 101).

SMARCAL1 is a multi-domain protein of the SNF2 family of ATPases (102). It associates with the active replisome complex and drives the remodeling of stalled forks by branch migration and fork regression. SMARCAL1-mediated remodeling has been shown to prevent an alternative repair mechanism involving the initial formation of double-strand breaks by MUS81 cleavage of the stalled fork (94).

Another member of the SNF2 family of remodelers is the ZRANB3 translocase. Upon induction of replication stress, ZBRANB3 associates with polyubiquitinated PCNA to facilitate RF reversal and replication slowdown (100). Ciccia et al. (103) showed that ZRANB3 activity is also required for resolution of recombination intermediates and efficient restart of arrested forks. In mammalian cells, siRNA-mediated downregulation of ZRANB3 leads to increased frequency of sister chromatid exchange and sensitivity of the cells to treatments interfering with replication, such as hydroxyurea (HU), camptothecin (CPT), cisplatin, and UV irradiation (103).

HLTF, the last member of the SNF2-family known to be required for fork remodeling so far, was originally identified as a human homolog of the yeast template-switching protein Rad5 (104). The ancient and conserved HIRAN domain was shown to be crucial for the interaction of HLTF with 3′-ssDNA at RFs (105). Similar to Rad5 in yeast, HLTF also possesses a E3-ubiquitin ligase-containing RING domain, which facilitates the K-63-linked polyubiquitination of PCNA (104). HLTF RING mutants were shown to fail in promoting efficient fork reversal, likely due to impaired recruitment of the downstream remodeler ZBRANB3 and other factors that require polyubiquitinated PCNA for efficient association with stalled RFs (100, 106).

The interplay between various fork remodeling factors seems to be highly complex and is not fully understood yet.

Deficiencies in SMARCAL1, ZRANB3, or HLTF lead to enhanced replication stress, collapse of stalled RFs and chromosomal instability, which sensitizes these cells to a wide range of replication stress-inducing agents (99, 100, 107). Lower expression or truncating gene mutations of SMARCAL1, ZRANB3, and HLTF have also been linked to susceptibility to various types of cancer (108–113). Recently, Puccetti et al. (114) identified non-redundant functions of SMARCAL1 and ZRANB3 in alleviation of Myc oncogene-induced replication stress. The authors also showed that both alleles of SMARCAL1 and ZRANB3 are required for fork stabilization in Myc-overexpressing primary cells (114). However, SMARCAL1-, ZRANB3-, and HLTF-mediated fork remodeling also possess a threat to genome integrity in cells lacking functional BRCA1/2 by providing a substrate for unregulated extensive degradation of the regressed arms (72, 96, 97, 106). An overview of the factors described in this and the following chapters can be found in Table 2.

**Table 2:** Overview of several key players involved in RF metabolism.

**Factor**	**Enzymatic activity**	**Function in RF remodeling/chemoresistance and clinical evidence**	**References**	
RAD51	Recombinase	RF reversal/depletion restores RF stability in BRCA-deficient cells *in vitro*.	(70)	
RAD54	DNA translocase	Regulation of RF reversal and restoration through branch migration.	(115)	
SMARCAL1 (SWI/SNF-related matrix-associated actin-dependent regulator of chromatin subfamily A-like protein 1)	ATP-dependent annealing helicase (translocase)	RF reversal/depletion restores RF stability and confers chemo-, PARPi-resistance in BRCA-deficient cells *in vitro*. Low mRNA associated with reduced survival in BRCA1-mutant breast cancer.	(96, 106)	
ZRANB3 (Zinc finger Ran-binding domain-containing protein 3)	ATP-dependent annealing helicase and endonuclease (translocase)	RF reversal/depletion restores RF stability in BRCA1/2-deficient cells *in vitro*.	(97, 100, 106)	
HLTF (Helicase-like transcription factor)	ATP-dependent annealing helicase (translocase)/E3 ubiquitin ligase	RF reversal/depletion restores RF stability in BRCA1/2-deficient cells *in vitro*.	(106)	
FBH1 (F-box DNA helicase 1)	DNA helicase/translocase	RF reversal	(116)	
BLM (Bloom syndrome protein)	ATP-dependent DNA helicase	RF reversal and restart	(117, 118)	
RECQL5 (RecQ protein-like 5)	ATP-dependent DNA helicase	RF reversal	(119)	
FANCM (Fanconi anemia group M protein)	ATP-dependent translocase	RF reversal, restart and protection of stalled forks	(120–122)	
RADX (RPA-related, RAD51-antagonist on X-chromosome)	ssDNA-binding protein	Antagonizing RF reversal/depletion restores RF stability and confers chemo- and PARPi-resistance in BRCA2-deficient cells *in vitro*.	(98)	
CtIP (CTBP-interacting protein)	5′ flap endonuclease	RF processing, restart of stalled forks	(72, 123)	
MRE11 (Meiotic recombination 11)	3′->5′ exonuclease and endonuclease	RF processing/inhibition restores RF stability in BRCA1/2-deficient cells *in vitro*.	(63, 72, 97, 124)	
RAD52		Recruitment of MRE11 to stalled RFs and fork degradation in BRCA2-deficient cells/depletion or inhibition restores RF stability in BRCA2-defective cells *in vitro*.	(97)	
PTIP (PAXIP1—PAX-interacting protein 1)		RF processing via recruitment of MRE11/loss restores RF stability *in vitro*. Poor prognosis in BRCA1/2 mutant ovarian cancer.	(62)	
PARP1 (Poly (ADP-ribose) polymerase 1)	Poly-ADP-ribosyltransferase	Recruitment of MRE11 to stalled RF, fork reversal, regulation of fork restart/deletion restores RF stability in BRCA1/2-deficient cells *in vitro*. Deficiency reduces tumor-free survival in Brca2^−/−^ mouse model.	(62, 125)	
EXO1 (Exonuclease 1)	5′->3′ exonuclease, 5′ structure specific DNA endonuclease, 5′->3′ RNase H	Further RF processing initiated by CtIP and MRE11/depletion restores RF stability in BRCA1/2-deficient cells *in vitro*.	(72)	
RECQ1 (ATP-dependent DNA helicase Q1)	ATP-dependent DNA helicase	RF restart via branch migration	(30)	
WRN (Werner syndrome ATP-dependent helicase)	ATP-dependent DNA helicase, 5′->3′ exonuclease	RF processing and HR-mediated restart of stalled forks	(126)	
DNA2 (DNA replication ATP-dependent helicase/nuclease)	ssDNA-dependent ATPase, 5′->3′ helicase, 5′->3′ endonuclease	RF processing and HR-mediated restart of stalled forks	(127)	
MUS81 (Methyl methanesulfonate and ultraviolet-sensitive gene clone 81)	Crossover junction endonuclease	RF fork processing and restart/Impaired recruitment via EZH2 inhibition or depletion restores RF stability in BRCA2-deficient cells *in vitro*. Low expression associated with poor prognosis in BRCA2-mutated tumors.	(73)	
CHD4 (Chromodomain-helicase-DNA-binding protein 4)	Chromatin remodeler	RF processing via chromatin accessibility/depletion restores RF stability in BRCA-deficient cells and confers chemoresistance *in vitro*. Poor prognosis in BRCA2 mutant ovarian cancer.	(62, 71)	
EZH2 (Enhancer of zeste homolog 2)	Chromatin modifier (Histone-lysine N-methyltransferase)	RF processing and restart via H3K27 trimethylation and MUS81 recruitment/depletion restores RF stability and confers chemoresistance in BRCA2-deficient cells. Low expression associated with poor prognosis in BRCA2-mutated tumors.	(73)	

## Mechanisms of Fork Restart

The ability to restart stalled RFs is essential to avoid excessive accumulation of replication intermediates, which are prone to aberrant processing and if not resolved properly, may cause chromosome segregation defects later in mitosis (128–130). To carry out this task, eukaryotic cells have evolved various mechanisms to process stalled replication intermediates and to restart affected RFs (Figure 2B, Table 2). Conversion of reversed forks back to standard three-way DNA junctions is a process essential for restoration of replication and successful duplication of the genome. In eukaryotes, failure in restarting severely damaged forks can be, to a certain extent, buffered by firing of dormant replication origins. However, systemic dysregulation of the process e.g., by genetic alterations or drug interventions significantly elevates chromosomal instability (131, 132).

RECQ1 is the most abundant member of the RecQ family of helicases in human cells (133, 134). However, its specific role in replication was not known for a long time. Thangavel et al. (134) showed that RECQ1 associates with replication origins in a cell cycle-dependent manner and that depletion of RECQ1 suppresses the RF rate in unperturbed S phase. Berti et al. (30) provided a mechanistic explanation for this phenotype by identifying the role of RECQ1 in priming branch migration at reversed forks and driving their restart (Figure 2B). By combining electron microscopy with single-molecule DNA fiber assay, Berti at al. (30) demonstrated a critical function of the RECQ1 helicase in promoting RF restart following topoisomerase 1 inhibition. Furthermore, the authors showed that the activity of RECQ1 at the reversed RFs is negatively regulated by PARP1, demonstrating a major role of PARylation in preventing RECQ1-mediated restart of forks.

Germline mutations leading to loss of the helicase activity of RECQ1 have been associated with increased susceptibility to breast cancer (135, 136). Another study showed that embryonic fibroblasts from mice lacking RECQ1 activity display increased rates of spontaneous chromosomal breakage and aneuploidy (132). Importantly, while genetic alterations reducing the activity of RECQ1 have been shown to increase susceptibility to certain types of cancer, overexpression of RECQ1 has been associated with increased replication stress survival, drug resistance, and overall poor prognosis in patients with multiple myeloma. The authors also showed that reducing RECQ1 expression by DNA methyltransferase inhibition sensitized multiple myeloma cells to PARPi (137). Collectively, these findings highlight the importance of RECQ1 in DNA metabolism and maintenance of chromosomal integrity and may open opportunities for novel targeted therapies (135, 136).

Another mechanism by which reversed RFs can be restarted involves unwinding of nascent strands in regressed arms by the ATP-dependent helicase activity of Werner syndrome protein (WRN) and nucleolytic processing by DNA2 (Figure 2B). Compared to other factors acting at stalled RFs, the role of WRN is more complex due to its dual helicase and exonuclease activities (126). Recruitment of WRN to reversed RFs and its proper function is highly dependent on an orchestrated action of ATM and ATR kinases. Interestingly, phosphorylation mediated by ATM and ATR is required for different steps in the process of stalled fork recovery. While ATR-mediated phosphorylation of multiple residues at the C-terminus of WRN is required for proper nuclear foci formation and co-localization with RPA, ATM-mediated phosphorylation is essential for formation of RAD51 nuclear foci, enabling proper recovery of collapsed forks (138). Furthermore, both helicase and exonuclease activities are required to limit MUS81-dependent breakage of forks after HU-induced arrest (126). Rodriguez-Lopez et al. (139) showed that normal progression RFs is affected in cells lacking functional WRN protein. The authors observed asymmetric progression of bi-directional forks diverging from the majority of replication origins, suggesting an increased frequency of RF stalling. Based on these data, the authors concluded that WRN is either protecting RFs from collapse or promotes resolution of replication intermediates at collapsed forks (139).

DNA2, like WRN, possesses nucleolytic and helicase activities. Together with exonuclease 1 (EXO1), DNA2 has been known for its function in mediating processive DSB resection downstream of the MRN complex and CtIP in eukaryotic cells. By nucleolytic processing of 5′ ends and generating 3′ ssDNA overhangs at DSBs, EXO1 and DNA2 carry out the initial step essential for HR (140–142). Independently of its role in dsDNA break repair, DNA2 has also been shown to assist WRN in controlling HR-mediated restart of reversed RFs by resecting the regressed arm following nucleotide depletion by HU (127). Importantly, this function of DNA2 may play a major role in tolerance to chronic replication stress, induced e.g., by oncogene activation, commonly exhibited by cancer cells. Indeed, Peng et al. (143) demonstrated that normal pancreatic ductal cells that were transformed into cancer cells by activating K-RAS showed overexpression of DNA2 in early stages of transformation. Elevated levels of DNA2 mRNA were also found in a wide range of cancer types, further demonstrating the importance of DNA2-mediated recovery of stalled forks in replication stress tolerance (143, 144).

The restart of reversed RFs via RECQ1- and DNA2/WRN-dependent pathways allows the resolution of most of the reversed RFs in S phase and is essential for maintenance of chromosomal integrity in eukaryotic cells (30, 127). Nevertheless, more processing is required in certain situations to prevent potentially mutagenic genomic rearrangements arising from unresolved complex replication intermediates (145). MUS81 is a cell-cycle regulated, structure-specific endonuclease that preferentially cleaves branched DNA substrates, such as replication or recombination intermediates. Processing of the reversed forks by MUS81 leads to formation of DSBs and subsequent recovery of stalled forks via HR (Figure 2B). MUS81-dependent processing of stalled forks was initially implicated in the resolution of forks perturbed by nucleotide pool depletion (146). However, other groups showed that processing of unusual replication intermediates by MUS81 may also be responsible for oncogene-induced genotoxicity, since depletion of MUS81 alleviated chromosomal breakage and resulted in an increase of reversed forks in human U2OS cells overexpressing the oncogenes *Cyclin E* and *Cdc25A* (147). Therefore, the outcome of MUS81-mediated DNA processing and DSB induction at stalled forks is highly dependent on the genetic background and the context in which the replication intermediates are formed.

## Fork Stability as a Resistance Mechanism in BRCA-Deficient Tumors

**Figure 3:**
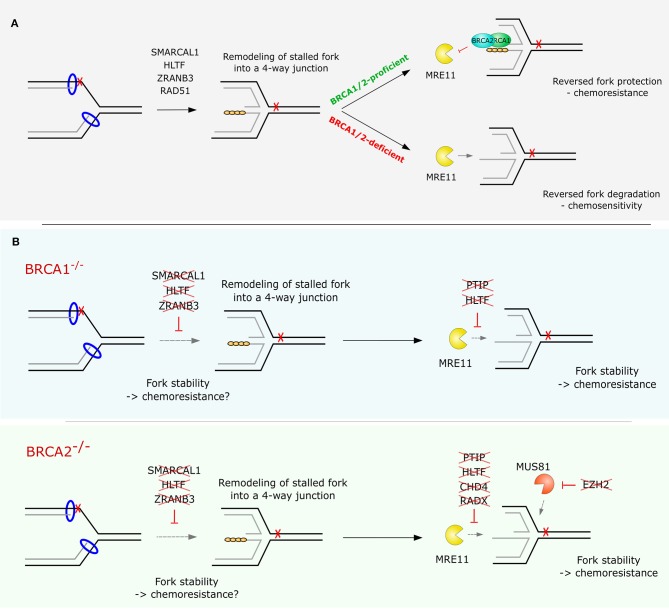
Replication fork stability or degradation in BRCA1/2-proficient and -deficient cells. **(A)** Reversed replication fork arms are protected from degradation by RAD51 nucleofilaments stabilized by BRCA1 and BRCA2. In the absence of BRCA1/2 proteins RAD51 dissociates from ssDNA at the regressed arms, leaving the nascent DNA susceptible to nucleolytic resection by exonucleases such as MRE11. **(B)** Overview of the factors shown to restore RF stability and confer chemoresistance upon their loss in BRCA1- or BRCA2-deficient cells.

BRCA1 and BRCA2 have well-known roles in the repair of DNA DSBs by HR. BRCA1 is crucial for the resection of DNA at DBS sites, creating two regions of ssDNA on either side of the break. BRCA2, with the help of PALB2, localizes the DNA recombinase RAD51 to the exposed ssDNA regions, forming stable nucleoprotein filaments which invade the intact homologous DNA double helix (148). Besides these, BRCA1/2 have many other cellular functions independent of their role in HR. One of these is their function in the protection of RFs under replication stress conditions by stabilizing RAD51 nucleofilaments and preventing excessive processing of forks by nucleases (Figure 3A) (63, 64, 149). While RF reversal has been shown to alleviate chromosomal instability upon exposure to genotoxic treatments (70), it also provides an entry point for nascent DNA degradation in cells lacking BRCA1 or BRCA2 (72, 96, 97, 106). Step-wise processing of nascent DNA at reversed forks by different nucleases has been shown to drive fork degradation. The MRE11-dependent resection is initiated by CtIP and then further extended by EXO1 (72). The enzymatic inhibition of MRE11 by mirin or siRNA-mediated depletion of EXO1 results in the protection of RFs in BRCA1/2-deficient cells treated with HU. Interestingly, the combination of MRE11 inhibition and EXO1 knockdown had a synergistic effect on the stability of stalled forks, indicating a potentially independent function of these nucleases in fork degradation (72). However, other groups have observed a full restoration of fork stability by MRE11 inhibition alone, pointing to MRE11 as the nuclease responsible for most of the processing of regressed arms in BRCA-deficient cells (62, 63). Furthermore, loss or down-regulation of factors involved in chromatin recruitment of MRE11 also restores fork stability and alleviates chromosome breakage in HU-treated BRCA-deficient cells (62).

Ray Chaudhuri et al. (62) showed that recruitment of MRE11 to stalled RFs is impaired upon loss of PTIP, a member of the MLL3/4 complex. The authors also demonstrated that *Ptip* deficiency rescues lethality in *Brca2*-deficient mouse embryonic stem cells. The restoration of RF stability promotes resistance of BRCA2-deficient tumors to cisplatin and PARPi independently of HR restoration. Interestingly, this function of PTIP at RFs is independent of its interaction with 53BP1 in the canonical DSB repair, since 53BP1/BRCA1-deficient B cells did not show any protection of forks upon nucleotide depletion (62). Similarly, loss of PARP1, which has been linked to regulation of MRE11-dependent restart and recombination at stalled forks (150), also restores RF stability and rescues lethality of *Brca2* null mouse embryonic stem cells (62). Another group demonstrated that depletion of RAD52, similarly to loss of PARP1 or PTIP, leads to reduced recruitment of MRE11 to chromatin and completely abolishes RF degradation in BRCA2-defective cells (97).

A genome-wide short hairpin RNA (shRNA) screen performed by Guillemente et al. (71) has identified the chromatin remodeling factor CHD4 to promote cisplatin resistance in *BRCA2*-mutated ovarian cancer cell line PEO-1 upon its downregulation. The depletion of CHD4 restored normal cell cycle progression and alleviated chromosomal aberrations upon cisplatin treatment (71). Mechanistically, similar to the situation in PTIP-,PARP1-, or RAD52-deficient cells, the phenotype of CHD4-depleted cells can be explained by the reduced chromatin recruitment of MRE11 and an increased RF stability in BRCA2-deficient cells upon replication stalling (62).

Various epigenetic modifications may also play an important role in RF remodeling and resolution of stalled RFs. Rondinelli et al. (73) performed a gene expression analysis of chromatin modifiers in HR-defective BRCA1/2-deficient tumors and found the enhancer of zeste homolog 2 (EZH2) to score as the top overexpressed chromatin modifier in various tumor types. The authors showed that EZH2 localizes to RFs stalled by HU and promotes recruitment of the MUS81 nuclease by mediating trimethylation of H3K27 (73). MUS81-dependent processing of stalled RFs has been shown to have a significant role in resolution of replication intermediates and replication restart (145, 151). Lai et al. proposed a new function of MUS81-dependent processing in replication stress tolerance and survival of BRCA2-deficient cells upon nucleotide depletion by HU. Lemacon et al. (72) then provided a mechanistic explanation for this phenotype by demonstrating that MUS81 resection at replication intermediates drives POLD3-dependent fork rescue upon HU-induced fork stalling. Interestingly, impaired MUS81 recruitment to RFs, e.g., by enzymatic inhibition or siRNA-mediated knockdown of EZH2, conferred RF stability and chemoresistance to PARPi and cisplatin in BRCA2-, but not in BRCA1-deficient cells (73). Consistent with these findings, low expression of EZH2/MUS81 have been found to correlate with chemoresistance and poor therapy outcome in patients with BRCA2-mutated tumors (73). However, it is not fully understood how MUS81 loss promotes PARPi resistance in BRCA2-deficient cells. The treatment-specific response of MUS81-depleted BRCA2-deficient cells to HU and PARPi may be explained by the importance of PARP1 in RF slowing and regulation of restart (30). Inhibition of PARP1 may promote RECQ1-dependent restart of reversed forks, therefore depriving cells of a substrate for MUS81 (30, 152, 153). However, more research has to be done to fully understand the context-specific synthetic lethal/viable interaction between BRCA2 and MUS81 deficiency.

Recently, the loss of RADX was identified as another mechanism protecting aberrant processing at stalled forks in BRCA2-deficient cells. RADX is an ssDNA binding protein that acts as a negative regulator of RAD51 (98). Dungrawala et al. (98) showed that inactivation of RADX enables excessive accumulation of RAD51 at RFs, leading to lower rate of replication elongation and formation of DSBs. However, in cells lacking BRCA2, depletion of RADX was sufficient to compensate for the decreased stability of RAD51 filaments and to rescue RF stability. This translated into reduced sensitivity to HU, cisplatin, CPT and PARPi.

Besides the proteins described above, several other factors have also been shown to promote RF remodeling such as DNA helicases FBH1, WRN, BLM, RECQL5, and DNA translocases RAD54 and FANCM (Table 2). However, the relevance of these proteins for replication fork metabolism in the context of BRCA1/2 deficiency and chemoresistance remains to be studied in more detail (115–117, 119, 120, 126). Collectively, genetic alterations resulting in rewired fork protection in BRCA1/2-deficient cells are highly complex and the interaction dynamics between various remodelers, processing factors, and other DNA repair factors remain to be further investigated. Furthermore, while loss of certain factors, such as PTIP, PARP1 (62), or fork remodelers SMARCAL1, HLTF, and ZRANB3 confer RF stability in both BRCA1- and BRCA2-deficient backgrounds (106), loss of CHD4, EZH2, and RADX only restore fork stability in cells lacking BRCA2 (Figure 3B) (71, 73, 98). These findings suggest that different pathways leading to restored fork stability may exist in mammalian cells, even though they all lead to the same endpoint: limited processing of stalled forks by nucleases (60). Importantly, while preventing reversed fork degradation by limiting nuclease access or activity (by loss of PTIP, CHD4, etc.) is likely to support therapy survival in the clinics, the possible impact of preventing formation of the reversed RF as a targeted structure for degradation is more debated.

## DNA Damage Tolerance Pathways

**Figure 4:**
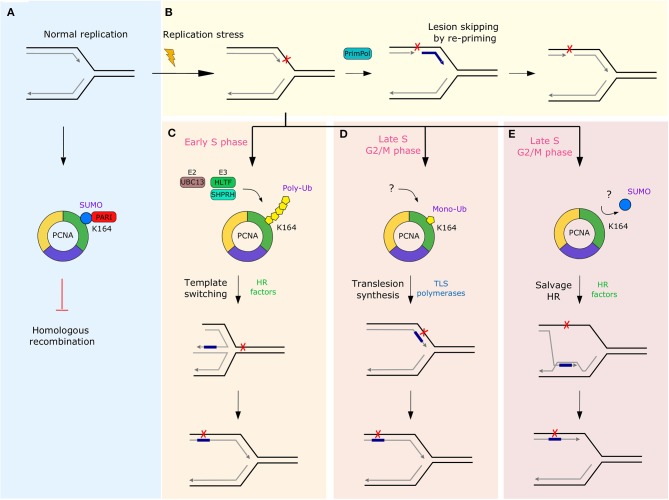
Overview of the DDT pathways and their regulation by various post-translational modifications of PCNA. **(A)** During normal replication PCNA interacts with the anti-recombinase PARI through SUMO modification to prevent potentially mutagenic recombination events in the absence of replication stress. **(B)** In response to replication stress, PrimPol-mediated lesion skipping allows cells to re-initiate synthesis downstream of the lesion and prevent RF stalling, while leaving an ssDNA gap behind. Alternatively, cells can employ one of three DDT pathways regulated by various modifications at K164 of PCNA. **(C)** Poly-ubiquitination in early S-phase initiates a mechanistically complex, but error-free TS, which requires RAD51-mediated strand invasion and newly replicated sister chromatid for synthesis over the damaged template. In contrast, mono-ubiquitination leads to the frequently mutagenic TLS in late S or G2/M phase. This process requires a step-wise exchange of high-fidelity replicative polymerases for specialized low-fidelity non-processive polymerases to enable synthesis over the lesion **(D)**. **(E)** The last DDT mechanism is “salvage” HR repair which is commonly repressed by SUMOylation of PCNA and by the anti-recombinase PARI in order to prevent chromosome rearrangements caused by hyper-recombination. The question marks indicate that the factors involved in the processes in human cells are not clearly defined. HR, homologous recombination; PARI, PCNA-associated recombination inhibitor; SUMO, small ubiquitin-like modifier; TLS, translesion synthesis; Ub, ubiquitin.

Another group of mechanisms allowing maintenance of genome integrity, which can involve RF remodeling, are DDT pathways. While the highly complex DDR network is essential for ensuring genome integrity over generations, immediate activation of the repair machinery at the damaged DNA may not be beneficial in every scenario. Prolonged stalling of RFs induced by DNA damage significantly increases the risk of fork collapse and genome instability. To minimize the chances of increased rates of fork collapse and formation of highly cytotoxic DSBs, cells developed DDT pathways that enable DNA synthesis beyond the damaged template, thereby completing the DNA replication prior to damage repair. The bypassed lesion is then removed later on by the specialized DNA repair pathways in the process called post-replicative repair (154, 155). Four major DDT pathways enabling bypass of DNA lesions have been described thus far: translesion synthesis (TLS), DNA primase-polymerase (PrimPol) mediated re-priming, template switching (TS) and the HR-mediated “salvage” pathway (156, 157) (Figure 4).

TLS is a mechanistically straightforward pathway compared to the TS and HR salvage repair, and it only requires the replacement of high-fidelity replicative polymerases by specialized low-fidelity non-processive polymerases (158). Low-fidelity of the TLS polymerases can be attributed to the lack of proofreading activity and the more flexible structure of the active site, which is able to accommodate modified bases and allow base mismatches (159, 160). Exchange of a stalled replicative polymerase for a TLS polymerase is a stepwise process involving at least two switching events (161). In the first step, the replicative polymerase is replaced by one of the insertion TLS polymerases, such as POL κ, POL ι, POL η, or REV1 that enable DNA synthesis over the DNA lesion. Then, either the same or another extension TLS polymerase elongates the newly synthesized DNA fragment to prevent detection of the lesion by the proof-reading activity of the replicative DNA polymerase (162, 163). This step is facilitated by the POL ζ complex of B-family polymerases (REV3L, REV7, POLD2, POLD3 (164–167). The last switching event restores a replicative DNA polymerase on the DNA template and reinitiates normal DNA synthesis. However, while the TLS is an easy, straightforward mechanism allowing lesion bypass and preventing fork stalling, it is also intrinsically error-prone. This is due to the higher frequency of nucleotide misincorporation by the TLS polymerases on the undamaged template, and due to the fact that synthesis over certain lesions, such as abasic sites, is often mutagenic (159, 160).

Another DDT mechanism is facilitated by the TLS primase PrimPol. PrimPol is a member of the archeo-eukaryotic primase (AEP) superfamily and has been shown to enable the bypass of various types of DNA lesions, either via its TLS activity or by lesion skipping (157, 168–171). While TLS is characterized by continuous DNA synthesis over the damaged template, lesion skipping involves the re-initiation of DNA synthesis of the leading strand *de novo* downstream of the replication block on the undamaged template. Therefore, PrimPol-mediated re-priming also represents a powerful RF remodeling-independent restart mechanism for stalled forks (172–175). Unlike TLS, lesion skipping results in the formation of a ssDNA gap behind the site of re-initiation and it needs to be repaired post-replicatively (170). PrimPol shares several properties with other TLS polymerases; it lacks the 3′-5′ exonuclease proofreading activity and exhibits low-fidelity and low-processivity DNA synthesis (157, 176–178).

Interestingly, experimental data from yeast and human cells indicate that DNA re-priming and stalled RF reversal are mutually exclusive events (175, 179). Disturbing the balance between fork reversal and re-priming may have a significant impact on genome stability maintenance, especially in the context of anticancer therapy in *BRCA1/2*-mutated tumors. Recent work of Quinet et al. demonstrated that the ATR-mediated increase in expression of PrimPol and its recruitment to stalled RFs abolishes the nascent DNA degradation in BRCA1/2-deficient human cells treated with multiple doses of genotoxic agents, such as UVC, HU and cisplatin. The authors also showed that the PrimPol-mediated adaptive response is dependent on ATR signaling. However, while elevated levels of Prim Pol lead to stalled RF protection, it also resulted in accumulation of ssDNA gaps in the genome (175). More research is required to fully understand the dynamics between the two pathways and the biological consequences of preventing RF degradation in BRCA-mutated tumors at the expense of accumulation of ssDNA gaps resulting from discontinuous replication.

Another, genetically distinct DDT pathway, TS, is a mechanistically more complex pathway for lesion bypass. In contrast to TLS, it uses the homologous template for synthesis, and therefore, facilitates an error-free synthesis over the damage site. Similarly to HR DNA repair, the initial step requires the stalled nascent strand to invade the newly replicated sister chromatid and is facilitated by RAD51 (156, 180, 181). The structure formed when the stalled nascent strand invades the undamaged chromatid is called the sister chromatid junction (SCJ). The undamaged template is then used to replicate DNA over the lesion containing the parental strand. After the gap is filled, SCJ is resolved back into two duplex DNA strands and the lesion bypass process is completed (156).

The last known DDT mechanism called “salvage” HR pathway is an alternative to the TS pathway. Like TS, salvage HR repair also employs template switching to bypass the DNA lesion. However, the major difference between the two pathways is that salvage HR repair is hyper-recombinogenic and thus only serves as the last resort of cells to replicate DNA over lesion if TLS and TS fail (182–184).

A tight regulation of pathway choice between the DDT mechanisms is important to limit the accumulation of mutations in case of TLS. It also prevents aberrant recombination events leading to potential genomic rearrangements and genome instability in the case of the salvage HR pathway. The regulation of the TLS, TS, and salvage HR pathways is facilitated by post-translational modifications (PTM) of PCNA (see Figure 4), which act as a molecular switch regulating pathway choice (185). In contrast to other pathways, PrimPol-mediated lesion bypass is not stimulated by PCNA and its PTMs. Instead, human PrimPol may be directly recruited to the stalled RFs through its interaction with the ssDNA-binding protein RPA (176). The initial PCNA modification, which is induced upon contact of a RF with the DNA lesion, is mono-ubiquitination at K164. In yeast, this modification is carried out by the E2-E3 complex Rad6-Rad18. In humans, however, several proteins seem to be implicated and their dynamics is not fully understood yet (186). Preferentially, the mono-ubiquitin mark would be extended to a poly-ubiquitin chain in a UBC13-dependent manner to stimulate ZRANB3-driven RF reversal and the error-free TS pathway in early S-phase (100, 103, 187, 188). In human cells, at least two E3 ubiquitin ligases can cooperate with UBC13 in promoting PCNA polyubiquitination; HLTF and SHPRH (104). However, their relative contribution to extending the mono-ubiquitin mark on PCNA is not well-understood yet. The second DDT pathway choice is the mutagenic TLS that has been shown to occur in late S or G2/M phase of the cell cycle. This pathway is initiated if the K164 mono-ubiquitin mark on PCNA is not extended (156, 189). The last choice is the salvage HR pathway, which is ubiquitin-independent. In yeast, the pathway is actively suppressed during normal S phase by sumoylation of PCNA at K164 and by the activity of the Srs2 anti-recombinase associated with SUMO-modified PCNA (190–192). In contrary to ubiquitination, sumoylation of PCNA is cell-cycle dependent and is strictly limited to S phase (193). Thus, HR-mediated lesion bypass is limited to late S and G2/M phases and serves only as the last resort for synthesis over the lesions that escaped the TS and TLS pathways (187). In humans, the Srs2 ortholog PARI (PCNA-associated recombination inhibitor) was shown to interact with PCNA and restrict unscheduled HR at RFs *in vitro* (194). However, the role of PCNA SUMOylation and its regulation in human cells is still debated (190).

## Alterations of DDT Pathways in Cancer

Defects in DNA replication or repair play a major role in genomic instability, one of the hallmarks of cancer. Given the importance of DDT pathways in the resolution of replication stress by preventing fork stalling and collapse, it is not surprising that alterations in genes encoding TLS polymerases and other DDT components have been associated with cancer development and drug resistance (195). When analyzing samples from various types of tumors, Albertella et al. found that about half of the tumor samples studied showed more than a 2-fold increase in expression of at least one specialized TLS DNA polymerase (196). On the one hand, increased activity of TLS polymerases may significantly contribute to mutagenicity and may increase the chances of oncogenic transformation (197). On the other hand, cancer cells with higher expression of these polymerases, such as Pol β, may escape the cytotoxic effect of various drugs, including alkylating agents, and hence significantly contribute to chemoresistance (198–200). Interestingly, different TLS polymerases were shown to be upregulated in different types of tumors; upregulation of Pol theta (Pol θ, POLQ) was shown to indicate poor outcome in breast cancer patients (201), while elevated expression of Pol eta (Pol η, POLH) correlates with decreased survival of patients with non-small cell lung cancer (202) or metastatic gastric adenocarcinoma treated with platinum drugs (203).

The ability of TLS polymerases to carry out replication over DNA lesions induced by anti-cancer treatments and therefore increase survival of cancer cells makes them attractive targets for improving the efficacy of currently used chemotherapeutics. Nevertheless, developing compounds highly selective toward TLS polymerases has been very challenging, mainly due to common substrates and some interaction partners shared by TLS and replicative polymerases (e.g., PCNA). Moreover, while several small molecule inhibitors of TLS components have been discovered, none of them were shown to have activity *in vivo* (204). Examples comprise previously described selective inhibitors of REV7 (205), oxetanocin derivatives inhibiting Pol η (206), or small molecule compounds blocking the interaction between components of the Pol ζ complex (207). One example of a small inhibitor shown to be active *in vivo* is a recently described molecule JH-RE-06. The compound prevents mutagenic TLS by blocking REV1-REV7 interaction and therefore, inhibiting the recruitment of polymerase POL ζ. This was shown to suppress TLS-mediated mutagenicity induced by cisplatin *in vitro* and to sensitize tumors to cisplatin treatment *in vivo* (204).

Moreover, suppression of various TLS components has been associated with an improved response to DNA damaging agents, such as cisplatin in certain types of tumors. siRNA-mediated knockdown of REV1 or REV3L (the essential subunit of POL ζ) was shown to sensitize intrinsically resistant tumors to chemotherapy or to reduce the frequency of acquired resistance in relapsed tumors (208). Doles et al. (209) showed that in addition to the pronounced sensitivity of REV3-deficient tumors to cisplatin and improved survival of treated mice, REV3-deficient cells also displayed lower amounts of cisplatin-induced mutations potentially decreasing a risk for secondary mutations leading to acquired resistance (209). Similarly, the suppression of Rev1 was shown to decrease cisplatin- and cyclophosphamide-induced mutagenesis in a mouse model for B-cell lymphoma and to limit acquired cyclophosphamide resistance *in vitro* (155). Moreover, DDT-defective *Pcna*^*K*164*R*^ lymphoma and breast cancer lines were also hypersensitive to cisplatin (210).

In summary, both DNA repair and DDT pathways are important to prevent RF collapse and maintain genome integrity. Therefore, defects in proteins involved in these processes can lead to cancer and also affect the response of cells to different genotoxic agents, which reflects on drug sensitivity or resistance in the clinic. However, several aspects of the intricate relationship between DDR and DDT, as well as their interaction at the RF are still unclear and need to be further investigated.

## Future Directions in Predicting Therapy Response

**Figure 5:**
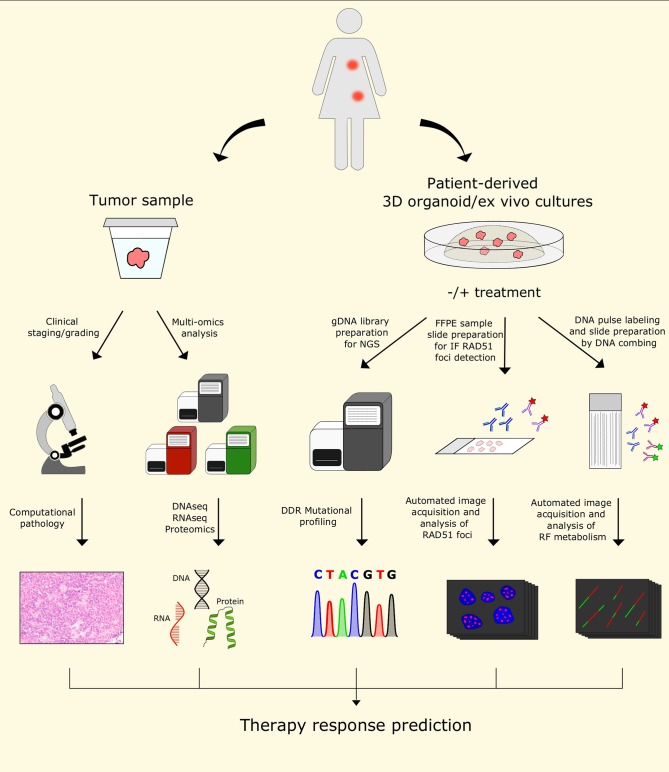
Future perspectives for predicting personalized therapy outcome. The use of patient samples for histology and multi-omics analysis will remain valuable tools to characterize tumors. In addition, patient-derived 3D organoid/*ex vivo* cultures may provide additional material for functional testing, such as RAD51 foci detection upon ionizing radiation, or DNA fiber analysis to probe for replication fork speed and/or stability. Together with the increasing knowledge of the importance of DDT and RF remodeling in anticancer drug response, these additional tools may allow automated functional analyses coupled with NGS profiling of DDR genes in patient-derived samples, providing the potential for designing personalized therapy strategies and predicting their outcomes in the future. DDR, DNA damage response; FFPE, formalin-fixed, paraffin-embedded; gDNA, genomic DNA; IF, immunofluorescence; NGS, next-generation sequencing; RF, replication fork.

The understanding of resistance mechanisms involving known DDR factors and/or RF remodelers/processors, together with the advance in biological *in vitro* and *in vivo* models for studying cancer, should be implemented in the clinical practice in the future for personalized diagnosis and for selecting an effective treatment strategy. Classical clinical and histopathological staging/grading will remain an import source of information. Here, we expect that computational pathology and deep learning algorithms will have a major impact to overcome the problem of inter-observer variability. Recent studies in breast cancer suggest that quantitative image analysis of histomorphometric features of early stage ER+ breast cancer are useful to predict patient survival independently (211, 212). Moreover, there are great expectations that the multiomics analysis of tumor samples, including next generation DNA/RNA sequencing, epigenomics, proteomics, and metabolomics, will make a difference to predict therapy response (Figure 5) [reviewed in (213–215)]. Indeed, the combination of these approaches has already been useful in exploring several aspects of the biological complexity of cancer (216, 217). However, some challenges in this context include the computational integration of such heterogeneous data and the availability of adequate amounts of optimally collected tumor tissue both before and during therapy.

Some novel computer tools are available for this type of integrated analysis [reviewed in (214)] and include platforms that analyse miRNA and mRNA expression (dChip-GemiNi, mirConnX, IntegraMiR), associate epigenomic with RNA expression and clinical data (such as MENT, MethHC, Wanderer, MethCNA) or integrate proteomic with several other types of data from multiple studies (XCMS Online, CancerSysDB) [reviewed in (214)].

The collection of data for multiomics analysis largely depends on the availability of patient samples. Moreover, the use of liquid biopsies and circulating tumor DNA for sequencing purposes would be complementary. Regarding the analysis at the protein level, the improvement in MS proteomics to reduce sample input and increase sensitivity for low abundance proteins would also help in this context.

The recent developments in the field of patient-derived 3D organoid cultures enable the expansion of tumor cells acquired by biopsy of different types of tumors (218–220). *In vitro*-cultured organoid lines often preserve morphological features, drug response profiles, as well as the heterogeneity of the original tumor (221). Therefore, 3D organoids could be another source of material for multi-omics approaches. However, it is important to keep in mind that the predictive power of tumor organoid cultures has clear limitations and is not 100% (222).

The ability to be rapidly expanded and genetically modified makes 3D organoids in principle a versatile tool for downstream functional testing of therapy response, including the study of RF biology (Figure 5) (62, 222). Nevertheless, the predictive power of 3D organoids has limitations that we still need to understand to make a significant step toward personalized medicine in clinical oncology (222). *Ex vivo* approaches to study living tumor fragments may be another direction in which RF biology in the context of anti-cancer therapy may be studied further.

Genetic testing for germline mutations in *BRCA1* and *BRCA2* has been available since the 1990s (223). Moreover, advances in next-generation sequencing (NGS) technology allowed for systematic investigation of the mutational landscape in *BRCA1-* and *BRCA2*-mutated tumors (224, 225). In addition, the identification of other DNA repair genes associated with HR deficiency opened the possibility for targeted therapy in those patients, including PARP inhibitors (226). Despite the undoubted significance of NGS data in predicting therapy success in patients with defects in the HR DNA repair pathway, this approach does not allow to study the role of epigenetics in modulating expression of HR genes, including *BRCA1* and *BRCA2*, nor functional testing for residual or restored HR repair or RF stability. Restoration of HR in BRCA1-deficient tumors by loss of 53BP1 is frequently found in tumors that acquire PARPi resistance (41, 50, 227). Similarly, loss of several other NHEJ and HR regulators, such as RIF1, REV7, and HELB have been shown to restore resection at DSB sites and promote HR repair, leading to improved DDT, chromosomal integrity, and consequently to acquired chemoresistance (44, 52, 53, 228). Restoration of damage-induced RAD51 foci formation is a well-established marker of DNA end processing and HR repair at DSBs. Therefore, implementing automated assays for RAD51 foci formation in patient samples would provide an important functional link to the complementary information acquired with next-generation sequencing on genetic alterations (Figure 5) (227).

As discussed above, the role of DDT pathways and DNA RF metabolism in the context of therapy response and resistance has gained a lot of attention in recent years. Various groups have identified novel factors implicated in the metabolism of DNA RFs and replication stress tolerance. Several of those factors, including DNA2, EZH2, and MUS81, showed the potential to be used as biomarkers for predicting response to DDR-targeting therapies in BRCA-deficient tumors (72, 137, 143). Nevertheless, similar to a functional HR restoration readout, functional assays for testing DDT, RF remodelers and fork stability would be needed to reliably phenotype tumor-derived samples and to predict therapy success. Recently, a novel system based on the formation of UVA-induced digoxygenin-tagged trimethylpsoralen ICLs was described by Mutreja et al. (86). Combined with the traditional DNA fiber spreading procedure, this technique allows the detection of individual ICL lesions and enables the study of cellular responses to ICL-inducing agents at the single-molecule resolution (86). One of the limitations of the DNA fiber technique currently used by many research groups is the time-consuming process of preparation of slides with the DNA spreads and the inter-observer variability of the image analysis (Table 1). Developing a pipeline for automated and standardized preparation of DNA fibers involving molecular combing and analysis of selected replication parameters, such as stability of stalled forks, rate of replication elongation, or lesion bypass, may enable a more precise prediction of therapy response in patients with DDR defects in their cancer. We hope that combining multiomics data with automated RAD51 foci formation and DNA RF analysis represents a powerful toolbox for predicting therapy outcome in patients with tumors defective in DDR pathways in the future (Figure 5).

## Author Contributions

ML and JB contributed equally to writting the manuscript and making the figures. SR contributed to writting and correcting the manuscript.

## Conflict of Interest

The authors declare that the research was conducted in the absence of any commercial or financial relationships that could be construed as a potential conflict of interest.
